# Supplementary material for: Public attitudes towards neurotechnology: Findings from two experiments concerning Brain Stimulation Devices (BSDs) and Brain-Computer Interfaces (BCIs)
Source: PLoS One. 2022 Nov 9;17(11):e0275454. doi: 10.1371/journal.pone.0275454 (PMC9645609; doi:10.1371/journal.pone.0275454)
Supplement: S1 File — (DOCX) [file pone.0275454.s001.docx]

Supplementary Information for the Manuscript “Public Attitudes towards Neurotechnology: Findings from Two Experiments Concerning Brain Stimulation Devices (BSDs) and Brain-Computer Interfaces (BCIs)”

## **Figure S1:** Vignettes examining use willingness and moral acceptability towards BSDs (Experiment 1).

| **Please read through the following carefully before answering the two questions:**   - Researchers are currently working on many new technologies. - Among these is the so-called “electrical brain stimulation,” for which *[INVASIVENESS: electrodes are fitted outside of the head, e.g., with a headband or a headset (see figure^1^). To control the impulse generator, a computer or a smartphone is used \| there is an operation whereby electrodes are fitted inside of the head (see figure^2^). A small battery-operated impulse generator with a computer chip as a controller is fitted under the skin of the upper body]*. - The electrodes produce weak electrical stimuli, thus activating or inhibiting the communication of nerve cells in different areas of the brain (for example the region for language or memory). - This technology can be used in the treatment of different illnesses (e.g., for chronic pain, epilepsy, Parkinson's disease, depression, and schizophrenia). - It can also be used when not medically required to improve mental performance (for example with concentration, memory and decision-making). - While this technology can have different benefits, there are also possible risks and side effects as well.   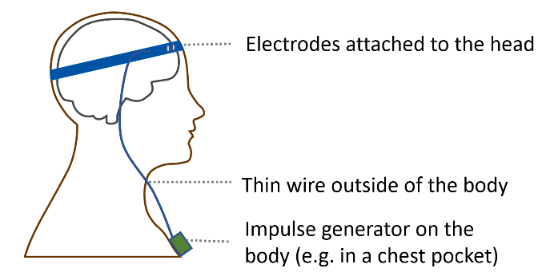  ^1^ Figure: Electrical brain stimulation  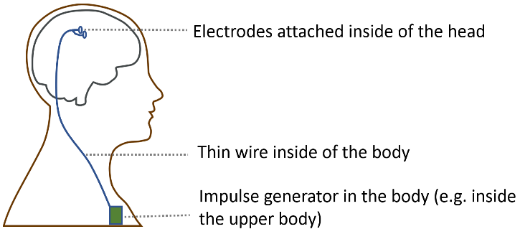  ^2^ Figure: Electrical brain stimulation | | | | | | |
| --- | --- | --- | --- | --- | --- | --- |
|  | | | | | | |
| **How would you evaluate the use of this technology for *[PURPOSE: medical reasons (e.g., for prevention, diagnosis, or treatment of an illness) \| non-medical reasons to improve one’s mental performance (e.g., in one’s spare time or profession)?]* Morally, I find it...^a^** | | | | | | |
| completely unacceptable |  |  |  | completely acceptable |  | no response |
| 0 | 1 | 2 | 3 | 4 |  |  |
| ○ | ○ | ○ | ○ | ○ |  | ○ |
|  | | | | | | |
| **Can you imagine using this technology for *[PURPOSE: medical reasons (e.g., for prevention, diagnosis, or treatment of an illness) \|without medical necessity for the improvement of your mental performance (e.g., in your spare time or profession)?]*^a^** | | | | | | |
| certainly not |  |  |  | certainly |  | no response |
| 0 | 1 | 2 | 3 | 4 |  |  |
| ○ | ○ | ○ | ○ | ○ |  | ○ |

*Note:* Italic text in brackets indicates the varied vignette dimensions (not italicized in the survey). ^a^The order pertaining moral acceptability and use willingness was randomized.

## **Figure S2:** Vignettes examining use willingness and moral acceptability towards BCIs (Experiment 2).

| **Please read through the following carefully before answering the two questions:**   - Researchers are currently working on many new technologies. - Among these are the so-called brain-computer interfaces, *[INVASIVENESS: with which electrodes are fitted outside of the head; it is worn like a bathing cap or baseball cap (see figure^1^) \| with which electrodes are fitted inside of the head by an operation, in a similar way to a pacemaker (see figure^2^)]*. - This technology facilitates communication between the brain and a computer. The electrodes measure activities in the brain (e.g., thoughts and emotions). This information is then processed by a computer. - Brain-computer interfaces can be used for medical purposes, e.g., for the prevention, diagnosis, and treatment of different illnesses (such as epilepsy and paralysis, for instance after a stroke). - This technology can also be used with no medical necessity, e.g., in one’s spare time or profession. - There are brain-computer interfaces with different functions. Some users can operate, for example, a prosthesis or a wheelchair with their thoughts, and also devices in the home, computer games, an internet browser, a car, or machines at work. Other brain-computer interfaces allow a person to convert thoughts into language or music. Some devices can give users feedback (e.g., through noises, optical stimuli, or a feeling). - While this technology can have different benefits, there are also possible risks and side effects as well.   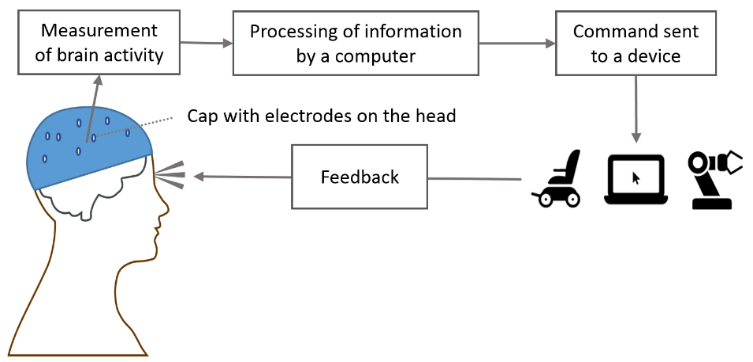  ^1^ Figure: Brain Computer Interfaces  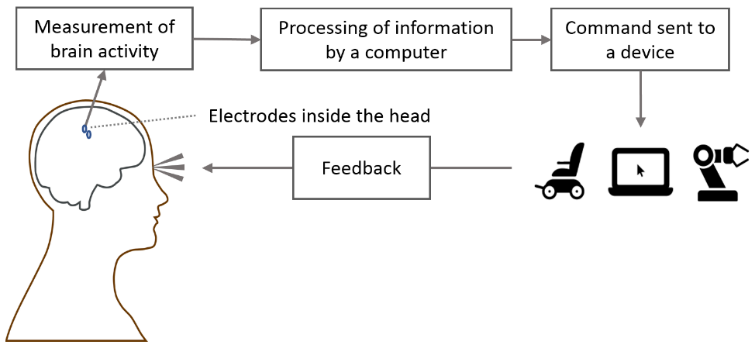  ^2^ Figure: Brain Computer Interfaces | | | | | | |
| --- | --- | --- | --- | --- | --- | --- |
|  | | | | | | |
| **How would you evaluate the use of this technology for *[PURPOSE: medical reasons (e.g., for prevention, diagnosis, or treatment of an illness) \| non-medical reasons to improve one’s mental performance (e.g., in one’s spare time or profession)?]* Morally, I find it...^a^** | | | | | | |
| completely unacceptable |  |  |  | completely acceptable |  | no response |
| 0 | 1 | 2 | 3 | 4 |  |  |
| ○ | ○ | ○ | ○ | ○ |  | ○ |
|  | | | | | | |
| **Can you imagine using this technology for *[PURPOSE: medical reasons (e.g., for prevention, diagnosis, or treatment of an illness) \|without medical necessity for the improvement of your mental performance (e.g., in your spare time or profession)?]*^a^** | | | | | | |
| certainly not |  |  |  | certainly |  | no response |
| 0 | 1 | 2 | 3 | 4 |  |  |
| ○ | ○ | ○ | ○ | ○ |  | ○ |

*Note:* Italic text in brackets indicates the varied vignette dimensions (not italicized in the survey). ^a^The order pertaining to moral acceptability and use willingness was randomized.

## **Figure S3:** Vignettes examining use willingness and moral acceptability towards BSDs – in the originally-used German language (Experiment 1).

| **Bitte lesen Sie das Folgende aufmerksam durch, bevor Sie die zwei Fragen beantworten:**   - Forscherinnen und Forscher arbeiten derzeit an vielen neuen Technologien. - Dazu zählt auch die sogenannte „elektrische Hirnstimulation“, für *[INVASIVENESS: die Elektroden außen am Kopf angelegt werden, z. B. mit einem Stirnband oder einem Headset (siehe Bild^1^). Zur Steuerung des Impulsgebers wird beispielsweise ein Computer oder ein Smartphone verwendet \| die bei einer Operation Elektroden in den Kopf eingesetzt werden (siehe Bild^2^). Zur Steuerung wird ein kleiner batteriebetriebener Impulsgeber mit einem Computerchip unter die Haut des Oberkörpers eingesetzt]* - Die Elektroden geben schwache elektrische Reize ab. So aktivieren oder hemmen sie die Kommunikation von Nervenzellen in verschiedenen Regionen des Gehirns (bspw. den Bereich für Sprache oder Gedächtnis). - Diese Technologie kann zur Behandlung verschiedener Krankheiten genutzt werden (z. B. bei chronischen Schmerzen, Epilepsie, Parkinson, Depression oder Schizophrenie). - Sie kann auch ohne medizinische Notwendigkeit genutzt werden, um die geistige Leistungsfähigkeit zu verbessern (wie z. B. die Konzentration, das Erinnerungsvermögen oder das Entscheidungsvermögen). - Während diese Technologie verschiedene Nutzen haben kann, so sind auch Risiken und Nebenwirkungen möglich.   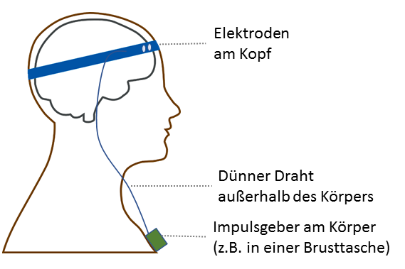  *^1^Bild: Elektrische Hirnstimulation*  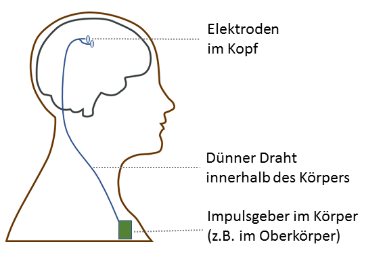  *^2^ Bild: Elektrische Hirnstimulation* | | | | | | |
| --- | --- | --- | --- | --- | --- | --- |
|  | | | | | | |
| **Wie bewerten Sie die Nutzung dieser Technologie aus *[PURPOSE: medizinischen Gründen (z. B. zur Vorbeugung, Diagnose oder Behandlung einer Krankheit) \| ohne medizinische Gründe zur Verbesserung der Leistung (z. B. in Freizeit oder Beruf)?]* Ich finde es moralisch…^a^** | | | | | | |
| völlig inakzeptabel |  |  |  | völlig  akzeptable |  | keine Angabe |
| 0 | 1 | 2 | 3 | 4 |  |  |
| ○ | ○ | ○ | ○ | ○ |  | ○ |
|  | | | | | | |
| **Können Sie sich vorstellen, diese Technologie *aus [PURPOSE: medizinischen Gründen zu nutzen (z. B. zur Vorbeugung, Diagnose oder Behandlung einer Krankheit)? \| ohne medizinische Notwendigkeit zur Verbesserung Ihrer Leistung zu nutzen (z. B. in Freizeit oder Beruf)?]*^a^** | | | | | | |
| ganz sicher nicht |  |  |  | ganz sicher |  | keine Angabe |
| 0 | 1 | 2 | 3 | 4 |  |  |
| ○ | ○ | ○ | ○ | ○ |  | ○ |

*Note:* Italic text in brackets indicates the varied vignette dimensions (not italicized in the survey). ^a^The order pertaining moral acceptability and use willingness was randomized.

## **Figure S4:** Vignettes examining use willingness and moral acceptability towards BCIs – in the originally-used German language (Experiment 2).

| **Bitte lesen Sie das Folgende aufmerksam durch, bevor Sie die zwei Fragen beantworten:**   - Forscherinnen und Forscher arbeiten derzeit an vielen neuen Technologien. - Dazu zählen auch die sogenannten „Brain-Computer-Interfaces“ (deutsch: Gehirn-Computer-Schnittstellen), *[INVASIVENESS: bei denen Elektroden außen am Kopf angelegt werden, dies wird getragen wie eine Bade- oder Baseballkappe (siehe Bild) \| für die bei einer Operation Elektroden in den Kopf eingesetzt werden, ähnlich wie bei einem Herzschrittmacher (siehe Bild)].* - Diese Technologie ermöglicht eine Kommunikation zwischen dem Gehirn und einem Computer. Dafür messen die Elektroden Aktivitäten im Gehirn (z. B. Gedanken und Emotionen). Diese Informationen werden dann von einem Computer verarbeitet. - Brain-Computer-Interfaces können für medizinische Zwecke angewendet werden, z. B. zur Vorbeugung, Diagnose oder Behandlung verschiedener Krankheiten (wie Epilepsie oder Lähmungen etwa nach einem Schlaganfall). - Die Technologie kann auch ohne medizinische Notwendigkeit angewendet werden, z. B. in Freizeit oder Beruf. - Es gibt Brain-Computer-Interfaces mit verschiedenen Funktionen. Mit manchen können Nutzerinnen und Nutzer mit ihren Gedanken z. B. eine Prothese oder einen Rollstuhl steuern, aber auch Geräte im Haushalt, Computerspiele, einen Internetbrowser, ein Auto oder Maschinen im Beruf. Andere Brain-Computer-Interfaces erlauben es Gedanken in Sprache oder Musik umzuwandeln. Einige Geräte können Nutzerinnen und Nutzer daraufhin Feedback geben (z. B. durch Geräusche, optische Reize oder ein Gefühl). - Während diese Technologie verschiedene Nutzen haben kann, so sind auch Risiken und Nebenwirkungen möglich.   *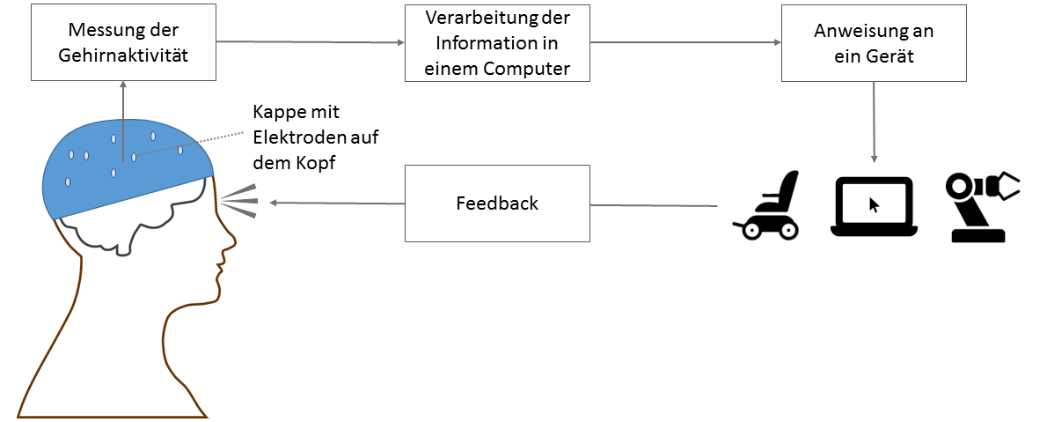*  *^1^Bild: Brain Computer Interfaces*  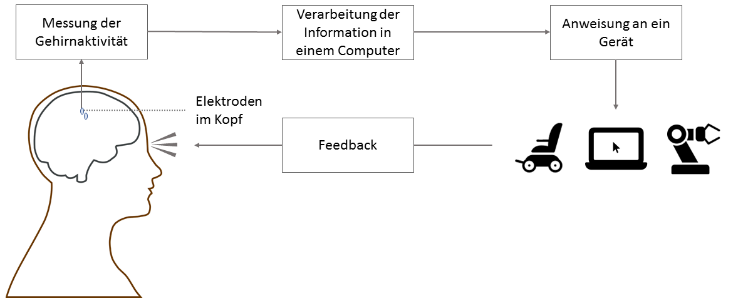  *^2^ Bild: Brain Computer Interfaces* | | | | | | |
| --- | --- | --- | --- | --- | --- | --- |
|  | | | | | | |
| **Wie bewerten Sie die Nutzung dieser Technologie aus *[PURPOSE: medizinischen Gründen (z. B. zur Vorbeugung, Diagnose oder Behandlung einer Krankheit) \| ohne medizinische Gründe zur Verbesserung der Leistung (z. B. in Freizeit oder Beruf)?]* Ich finde es moralisch…^a^** | | | | | | |
| völlig inakzeptabel |  |  |  | völlig  akzeptable |  | keine Angabe |
| 0 | 1 | 2 | 3 | 4 |  |  |
| ○ | ○ | ○ | ○ | ○ |  | ○ |
|  | | | | | | |
| **Können Sie sich vorstellen, diese Technologie *aus [PURPOSE: medizinischen Gründen zu nutzen (z. B. zur Vorbeugung, Diagnose oder Behandlung einer Krankheit)? \| ohne medizinische Notwendigkeit zur Verbesserung Ihrer Leistung zu nutzen (z. B. in Freizeit oder Beruf)?]*^a^** | | | | | | |
| ganz sicher nicht |  |  |  | ganz sicher |  | keine Angabe |
| 0 | 1 | 2 | 3 | 4 |  |  |
| ○ | ○ | ○ | ○ | ○ |  | ○ |

*Note:* Italic text in brackets indicates the varied vignette dimensions (not italicized in the survey). ^a^The order pertaining moral acceptability and use willingness was randomized.

## **Table S1:** Ordinary least regression of the moral acceptability regarding BSDs – testing for effects of the purpose of use (enhancement vs. treatment) conditional on each study variable (*N_Experiment 1_*=1,090).

|  | M_1_ | M_2_ | M_3_ | M_4_ | M_5_ | M_6_ | M_7_ | M_8_ |
| --- | --- | --- | --- | --- | --- | --- | --- | --- |
| Enhancement | -1.93^***^ | -2.16^***^ | -2.61^***^ | -2.48^***^ | -1.42^***^ | -1.98^***^ | -2.13^***^ | -2.09^***^ |
| (Ref. Treatment) | (0.10) | (0.10) | (0.15) | (0.14) | (0.24) | (0.10) | (0.10) | (0.09) |
| Invasive (Ref. | 0.01 | -0.21^**^ | -0.22^***^ | -0.22^**^ | -0.22^**^ | -0.21^**^ | -0.21^**^ | -0.21^**^ |
| Noninvasive) | (0.09) | (0.07) | (0.07) | (0.07) | (0.07) | (0.07) | (0.07) | (0.07) |
| Morality first | 0.09 | 0.08 | 0.09 | 0.08 | 0.08 | 0.09 | 0.08 | 0.09 |
| (Ref. Second) | (0.07) | (0.10) | (0.07) | (0.07) | (0.07) | (0.07) | (0.07) | (0.07) |
| Stress | 0.11^*^ | 0.10 | -0.05 | 0.11 | 0.10 | 0.11 | 0.10 | 0.10 |
|  | (0.06) | (0.06) | (0.07) | (0.06) | (0.06) | (0.06) | (0.06) | (0.06) |
| Low cognitive | -0.05 | -0.05 | -0.05 | -0.20^*^ | -0.05 | -0.05 | -0.05 | -0.05 |
| functioning | (0.06) | (0.06) | (0.06) | (0.08) | (0.06) | (0.06) | (0.06) | (0.06) |
| Age | -0.00 | -0.00 | -0.00 | -0.00 | 0.00 | -0.00 | -0.00 | -0.00 |
|  | (0.00) | (0.00) | (0.00) | (0.00) | (0.00) | (0.00) | (0.00) | (0.00) |
| Religiosity | -0.02^*^ | -0.02^*^ | -0.02^*^ | -0.02^*^ | -0.02^*^ | 0.00 | -0.02^*^ | -0.02^*^ |
|  | (0.01) | (0.01) | (0.01) | (0.01) | (0.01) | (0.01) | (0.01) | (0.01) |
| Secondary education | 0.14^*^ | 0.13 | 0.13 | 0.12 | 0.12 | 0.12 | 0.15 | 0.13 |
| (Ref. Lower) | (0.07) | (0.07) | (0.07) | (0.07) | (0.07) | (0.07) | (0.10) | (0.07) |
| Female | -0.05 | -0.05 | -0.06 | -0.07 | -0.05 | -0.05 | -0.05 | 0.01 |
| (Ref. Male) | (0.07) | (0.07) | (0.07) | (0.07) | (0.07) | (0.07) | (0.07) | (0.10) |
| Enhancement× | -0.44^**^ |  |  |  |  |  |  |  |
| Invasive | (0.13) |  |  |  |  |  |  |  |
| Enhancement× |  | 0.02 |  |  |  |  |  |  |
| Morality first |  | (0.13) |  |  |  |  |  |  |
| Enhancement× |  |  | 0.31^***^ |  |  |  |  |  |
| Stress |  |  | (0.09) |  |  |  |  |  |
| Enhancement× |  |  |  | 0.28^**^ |  |  |  |  |
| Low cog. funct. |  |  |  | (0.10) |  |  |  |  |
| Enhancement× |  |  |  |  | -0.01^**^ |  |  |  |
| Age |  |  |  |  | (0.00) |  |  |  |
| Enhancement× |  |  |  |  |  | -0.05^*^ |  |  |
| Religiosity |  |  |  |  |  | (0.02) |  |  |
| Enhancement× |  |  |  |  |  |  | -0.04 |  |
| Secondary education |  |  |  |  |  |  | (0.14) |  |
| Enhancement× |  |  |  |  |  |  |  | -0.12 |
| Female |  |  |  |  |  |  |  | (0.14) |
| Constant | 3.17^***^ | 3.30^***^ | 3.54^***^ | 3.49^***^ | 2.98^***^ | 3.20^***^ | 3.29^***^ | 3.27^***^ |
|  | (0.20) | (0.20) | (0.20) | (0.20) | (0.22) | (0.20) | (0.20) | (0.20) |
| *F-test* | 108.41^***^ | 106.27^***^ | 108.67^***^ | 107.72^***^ | 108.24^***^ | 107.34^***^ | 106.28^***^ | 106.53^***^ |

*Notes:* *B*-coefficients (standard errors in brackets); ^*^*p*<0.05, ^**^*p*<0.01, ^***^*p*<0.001.

## **Table S2:** Ordinary least regression of the use willingness regarding BSDs – testing for effects of the purpose of use (enhancement vs. treatment) conditional on each study variable (*N_Experiment 1_*=1,090).

|  | M_1_ | M_2_ | M_3_ | M_4_ | M_5_ | M_6_ | M_7_ | M_8_ |
| --- | --- | --- | --- | --- | --- | --- | --- | --- |
| Enhancement | -2.14^***^ | -2.05^***^ | -2.64^***^ | -2.56^***^ | -1.81^***^ | -2.18^***^ | -2.17^***^ | -2.26^***^ |
| (Ref. Treatment) | (0.09) | (0.09) | (0.14) | (0.13) | (0.22) | (0.09) | (0.09) | (0.08) |
| Invasive (Ref. | -0.20^*^ | -0.28^***^ | -0.29^***^ | -0.28^***^ | -0.28^***^ | -0.28^***^ | -0.28^***^ | -0.28^***^ |
| Noninvasive) | (0.09) | (0.06) | (0.06) | (0.06) | (0.06) | (0.06) | (0.06) | (0.06) |
| Morality first | 0.19^**^ | 0.35^***^ | 0.19^**^ | 0.19^**^ | 0.19^**^ | 0.19^**^ | 0.19^**^ | 0.19^**^ |
| (Ref. Second) | (0.06) | (0.09) | (0.06) | (0.06) | (0.06) | (0.06) | (0.06) | (0.06) |
| Stress | 0.08 | 0.07 | -0.07 | 0.08 | 0.07 | 0.07 | 0.07 | 0.07 |
|  | (0.05) | (0.05) | (0.07) | (0.05) | (0.05) | (0.05) | (0.05) | (0.05) |
| Low cognitive | -0.05 | -0.05 | -0.05 | -0.20^**^ | -0.05 | -0.05 | -0.05 | -0.05 |
| functioning | (0.06) | (0.06) | (0.06) | (0.07) | (0.06) | (0.06) | (0.06) | (0.06) |
| Age | -0.01^**^ | -0.01^**^ | -0.01^**^ | -0.01^**^ | -0.00 | -0.01^**^ | -0.01^**^ | -0.01^**^ |
|  | (0.00) | (0.00) | (0.00) | (0.00) | (0.00) | (0.00) | (0.00) | (0.00) |
| Religiosity | -0.01 | -0.01 | -0.02 | -0.02 | -0.01 | -0.01 | -0.01 | -0.01 |
|  | (0.01) | (0.01) | (0.01) | (0.01) | (0.01) | (0.01) | (0.01) | (0.01) |
| Secondary education | 0.04 | 0.04 | 0.04 | 0.03 | 0.03 | 0.04 | 0.09 | 0.04 |
| (Ref. Lower) | (0.06) | (0.06) | (0.06) | (0.06) | (0.06) | (0.06) | (0.09) | (0.06) |
| Female | -0.12 | -0.11 | -0.12 | -0.13^*^ | -0.12 | -0.11 | -0.11 | -0.16 |
| (Ref. Male) | (0.07) | (0.07) | (0.07) | (0.07) | (0.07) | (0.07) | (0.07) | (0.09) |
| Enhancement× | -0.15 |  |  |  |  |  |  |  |
| Invasive | (0.13) |  |  |  |  |  |  |  |
| Enhancement× |  | -0.32^*^ |  |  |  |  |  |  |
| Morality first |  | (0.13) |  |  |  |  |  |  |
| Enhancement× |  |  | 0.29^***^ |  |  |  |  |  |
| Stress |  |  | (0.08) |  |  |  |  |  |
| Enhancement× |  |  |  | 0.29^**^ |  |  |  |  |
| Low cog. funct. |  |  |  | (0.10) |  |  |  |  |
| Enhancement× |  |  |  |  | -0.01 |  |  |  |
| Age |  |  |  |  | (0.00) |  |  |  |
| Enhancement× |  |  |  |  |  | -0.01 |  |  |
| Religiosity |  |  |  |  |  | (0.02) |  |  |
| Enhancement× |  |  |  |  |  |  | -0.10 |  |
| Secondary education |  |  |  |  |  |  | (0.13) |  |
| Enhancement× |  |  |  |  |  |  |  | 0.09 |
| Female |  |  |  |  |  |  |  | (0.13) |
| Constant | 3.04^***^ | 3.01^***^ | 3.31^***^ | 3.28^***^ | 2.90^***^ | 3.06^***^ | 3.06^***^ | 3.11^***^ |
|  | (0.18) | (0.18) | (0.19) | (0.19) | (0.20) | (0.18) | (0.18) | (0.18) |
| *F-test* | 131.23^***^ | 132.32^***^ | 133.46^***^ | 132.93^***^ | 131.72^***^ | 130.97^***^ | 131.03^***^ | 131.01^***^ |

*Notes:* *B*-coefficients (standard errors in brackets); ^*^*p*<0.05, ^**^*p*<0.01, ^***^*p*<0.001.

## **Table S3:** Ordinary least regression of the moral acceptability regarding BCIs – testing for effects of purpose of use (enhancement vs. treatment) conditional on each study variable (*N_Experiment 2_*=1,089).

|  | M_1_ | M_2_ | M_3_ | M_4_ | M_5_ | M_6_ | M_7_ | M_8_ |
| --- | --- | --- | --- | --- | --- | --- | --- | --- |
| Enhancement | -1.91^***^ | -2.04^***^ | -2.21^***^ | -2.07^***^ | -1.42^***^ | -1.76^***^ | -1.87^***^ | -1.79^***^ |
| (Ref. Treatment) | (0.10) | (0.10) | (0.16) | (0.14) | (0.27) | (0.11) | (0.10) | (0.10) |
| Invasive (Ref. | -0.33^***^ | -0.35^***^ | -0.35^***^ | -0.35^***^ | -0.35^***^ | -0.35^***^ | -0.35^***^ | -0.35^***^ |
| Noninvasive) | (0.10) | (0.07) | (0.07) | (0.07) | (0.07) | (0.07) | (0.07) | (0.07) |
| Morality first | 0.17^*^ | 0.06 | 0.17^*^ | 0.18^*^ | 0.17^*^ | 0.17^*^ | 0.18^*^ | 0.18^*^ |
| (Ref. Second) | (0.07) | (0.10) | (0.07) | (0.07) | (0.07) | (0.07) | (0.07) | (0.07) |
| Stress | -0.02 | -0.03 | -0.12 | -0.02 | -0.03 | -0.02 | -0.02 | -0.02 |
|  | (0.06) | (0.06) | (0.07) | (0.06) | (0.06) | (0.06) | (0.06) | (0.06) |
| Low cognitive | 0.06 | 0.06 | 0.06 | -0.00 | 0.07 | 0.06 | 0.06 | 0.06 |
| functioning | (0.06) | (0.06) | (0.06) | (0.08) | (0.06) | (0.06) | (0.06) | (0.06) |
| Age | -0.01^***^ | -0.01^***^ | -0.01^***^ | -0.01^***^ | -0.01 | -0.01^***^ | -0.01^***^ | -0.01^***^ |
|  | (0.00) | (0.00) | (0.00) | (0.00) | (0.00) | (0.00) | (0.00) | (0.00) |
| Religiosity | -0.02^*^ | -0.02^*^ | -0.02^*^ | -0.02^*^ | -0.02^*^ | -0.00 | -0.02^*^ | -0.02^*^ |
|  | (0.01) | (0.01) | (0.01) | (0.01) | (0.01) | (0.02) | (0.01) | (0.01) |
| Secondary education | 0.11 | 0.10 | 0.11 | 0.11 | 0.11 | 0.11 | 0.16 | 0.11 |
| (Ref. Lower) | (0.07) | (0.07) | (0.07) | (0.07) | (0.07) | (0.07) | (0.10) | (0.07) |
| Female | -0.18^*^ | -0.18^*^ | -0.18^*^ | -0.18^*^ | -0.18^*^ | -0.18^*^ | -0.18^*^ | -0.03 |
| (Ref. Male) | (0.07) | (0.07) | (0.07) | (0.07) | (0.07) | (0.07) | (0.07) | (0.10) |
| Enhancement× | -0.04 |  |  |  |  |  |  |  |
| Invasive | (0.14) |  |  |  |  |  |  |  |
| Enhancement× |  | 0.22 |  |  |  |  |  |  |
| Morality first |  | (0.14) |  |  |  |  |  |  |
| Enhancement× |  |  | 0.19^*^ |  |  |  |  |  |
| Stress |  |  | (0.09) |  |  |  |  |  |
| Enhancement× |  |  |  | 0.13 |  |  |  |  |
| Low cog. funct. |  |  |  | (0.11) |  |  |  |  |
| Enhancement× |  |  |  |  | -0.01 |  |  |  |
| Age |  |  |  |  | (0.00) |  |  |  |
| Enhancement× |  |  |  |  |  | -0.04^*^ |  |  |
| Religiosity |  |  |  |  |  | (0.02) |  |  |
| Enhancement× |  |  |  |  |  |  | -0.10 |  |
| Secondary education |  |  |  |  |  |  | (0.14) |  |
| Enhancement× |  |  |  |  |  |  |  | -0.30^*^ |
| Female |  |  |  |  |  |  |  | (0.14) |
| Constant | 3.87^***^ | 3.94^***^ | 4.02^***^ | 3.96^***^ | 3.63^***^ | 3.79^***^ | 3.85^***^ | 3.80^***^ |
|  | (0.21) | (0.21) | (0.22) | (0.21) | (0.24) | (0.21) | (0.21) | (0.21) |
| *F-test* | 81.82^***^ | 82.25^***^ | 82.54^***^ | 82.05^***^ | 82.46^***^ | 82.54^***^ | 81.90^***^ | 82.58^***^ |

*Notes:* *B*-coefficients (standard errors in brackets); ^*^*p*<0.05, ^**^*p*<0.01, ^***^*p*<0.001.

## **Table S4:** Ordinary least regression of the use willingness regarding BCIs – testing for effects of purpose of use (enhancement vs. treatment) conditional on each study variable (*N_Experiment 2_*=1,089).

|  | M_1_ | M_2_ | M_3_ | M_4_ | M_5_ | M_6_ | M_7_ | M_8_ |
| --- | --- | --- | --- | --- | --- | --- | --- | --- |
| Enhancement | -2.12^***^ | -1.98^***^ | -2.39^***^ | -2.29^***^ | -1.68^***^ | -1.98^***^ | -2.06^***^ | -2.00^***^ |
| (Ref. Treatment) | (0.10) | (0.09) | (0.15) | (0.14) | (0.25) | (0.10) | (0.10) | (0.09) |
| Invasive (Ref. | -0.38^***^ | -0.35^***^ | -0.35^***^ | -0.34^***^ | -0.35^***^ | -0.35^***^ | -0.35^***^ | -0.35^***^ |
| Noninvasive) | (0.09) | (0.07) | (0.07) | (0.07) | (0.07) | (0.07) | (0.07) | (0.07) |
| Morality first | 0.21^**^ | 0.32^***^ | 0.20^**^ | 0.21^**^ | 0.21^**^ | 0.21^**^ | 0.21^**^ | 0.21^**^ |
| (Ref. Second) | (0.07) | (0.10) | (0.07) | (0.07) | (0.07) | (0.07) | (0.07) | (0.07) |
| Stress | 0.07 | 0.08 | -0.03 | 0.07 | 0.07 | 0.07 | 0.07 | 0.07 |
|  | (0.05) | (0.05) | (0.07) | (0.05) | (0.05) | (0.05) | (0.05) | (0.05) |
| Low cognitive | -0.03 | -0.03 | -0.03 | -0.11 | -0.02 | -0.03 | -0.03 | -0.03 |
| functioning | (0.06) | (0.06) | (0.06) | (0.08) | (0.06) | (0.06) | (0.06) | (0.06) |
| Age | -0.01^***^ | -0.01^***^ | -0.01^***^ | -0.01^***^ | -0.01^**^ | -0.01^***^ | -0.01^***^ | -0.01^***^ |
|  | (0.00) | (0.00) | (0.00) | (0.00) | (0.00) | (0.00) | (0.00) | (0.00) |
| Religiosity | -0.01 | -0.01 | -0.01 | -0.01 | -0.01 | 0.00 | -0.01 | -0.01 |
|  | (0.01) | (0.01) | (0.01) | (0.01) | (0.01) | (0.01) | (0.01) | (0.01) |
| Secondary education | 0.09 | 0.09 | 0.09 | 0.09 | 0.09 | 0.09 | 0.12 | 0.09 |
| (Ref. Lower) | (0.07) | (0.07) | (0.07) | (0.07) | (0.07) | (0.07) | (0.10) | (0.07) |
| Female | -0.22^**^ | -0.22^**^ | -0.22^**^ | -0.22^**^ | -0.21^**^ | -0.22^**^ | -0.22^**^ | -0.12 |
| (Ref. Male) | (0.07) | (0.07) | (0.07) | (0.07) | (0.07) | (0.07) | (0.07) | (0.10) |
| Enhancement× | 0.06 |  |  |  |  |  |  |  |
| Invasive | (0.13) |  |  |  |  |  |  |  |
| Enhancement× |  | -0.22 |  |  |  |  |  |  |
| Morality first |  | (0.13) |  |  |  |  |  |  |
| Enhancement× |  |  | 0.21^*^ |  |  |  |  |  |
| Stress |  |  | (0.09) |  |  |  |  |  |
| Enhancement× |  |  |  | 0.17 |  |  |  |  |
| Low cog. funct. |  |  |  | (0.10) |  |  |  |  |
| Enhancement× |  |  |  |  | -0.01 |  |  |  |
| Age |  |  |  |  | (0.00) |  |  |  |
| Enhancement× |  |  |  |  |  | -0.03 |  |  |
| Religiosity |  |  |  |  |  | (0.02) |  |  |
| Enhancement× |  |  |  |  |  |  | -0.06 |  |
| Secondary education |  |  |  |  |  |  | (0.13) |  |
| Enhancement× |  |  |  |  |  |  |  | -0.20 |
| Female |  |  |  |  |  |  |  | (0.13) |
| Constant | 3.68^***^ | 3.61^***^ | 3.81^***^ | 3.77^***^ | 3.46^***^ | 3.61^***^ | 3.65^***^ | 3.61^***^ |
|  | (0.20) | (0.20) | (0.20) | (0.20) | (0.23) | (0.20) | (0.20) | (0.20) |
| *F-test* | 107.93^***^ | 108.42^***^ | 108.96^***^ | 108.44^***^ | 108.46^***^ | 108.30^***^ | 107.94^***^ | 108.34^***^ |

*Notes:* *B*-coefficients (standard errors in brackets); ^*^*p*<0.05, ^**^*p*<0.01, ^***^*p*<0.001.

## **Table S5:** Ordinary least regression of the moral acceptability and use willingness regarding BSDs (*N_Experiment 1_*=1,083^a^) and regarding BCIs (*N_Experiment 2_*=1,080^a^) – controlled for anonymity perceptions.

|  | BSD:  Moral acceptability | BSD:  Use willingness | BCI:  Moral acceptability | BCI:  Use willingness |
| --- | --- | --- | --- | --- |
| Enhancement (Ref. Treatment) | -2.16^***^ | -2.22^***^ | -1.92^***^ | -2.08^***^ |
|  | (0.07) | (0.06) | (0.07) | (0.07) |
| Invasive (Ref. Noninvasive) | -0.21^**^ | -0.28^***^ | -0.36^***^ | -0.36^***^ |
|  | (0.07) | (0.06) | (0.07) | (0.07) |
| Morality first (Ref. Second) | 0.10 | 0.20^**^ | 0.18^*^ | 0.21^**^ |
|  | (0.07) | (0.06) | (0.07) | (0.07) |
| Stress | 0.11^*^ | 0.08 | -0.03 | 0.08 |
|  | (0.06) | (0.05) | (0.06) | (0.05) |
| Low cognitive functioning | -0.07 | -0.06 | 0.07 | -0.03 |
|  | (0.06) | (0.06) | (0.06) | (0.06) |
| Age | -0.00 | -0.01^**^ | -0.01^***^ | -0.01^***^ |
|  | (0.00) | (0.00) | (0.00) | (0.00) |
| Religiosity | -0.02^*^ | -0.02 | -0.02^*^ | -0.01 |
|  | (0.01) | (0.01) | (0.01) | (0.01) |
| Secondary education (Ref. Lower) | 0.14^*^ | 0.05 | 0.11 | 0.09 |
|  | (0.07) | (0.07) | (0.07) | (0.07) |
| Female (Ref. Male) | -0.03 | -0.10 | -0.17^*^ | -0.21^**^ |
|  | (0.07) | (0.07) | (0.07) | (0.07) |
| Anonymity perceptions | -0.02 | -0.01 | 0.02 | 0.01 |
|  | (0.05) | (0.05) | (0.05) | (0.05) |
| Constant | 3.39^***^ | 3.10^***^ | 3.77^***^ | 3.57^***^ |
|  | (0.29) | (0.28) | (0.30) | (0.29) |
| *F-test* | 107.79^***^ | 131.30^***^ | 80.97^***^ | 105.94^***^ |
| Adjusted *R*^2^ | 0.497 | 0.546 | 0.426 | 0.493 |

*Notes:* *B*-coefficients (standard errors in brackets); ^*^*p*<0.05, ^**^*p*<0.01, ^***^*p*<0.001. ^a^*N* lower than in Table 2 due to the missing values in the anonymity variable.

## **Table S6:** Ordinary least regression of the moral acceptability regarding BSDs – testing for effects of purpose of use (enhancement vs. treatment) conditional on each study variable (*N_Experiment 1_*=1,083^a^) – controlled for anonymity perceptions.

|  | M_1_ | M_2_ | M_3_ | M_4_ | M_5_ | M_6_ | M_7_ | M_8_ |
| --- | --- | --- | --- | --- | --- | --- | --- | --- |
| Enhancement | -1.94^***^ | -2.16^***^ | -2.60^***^ | -2.50^***^ | -1.41^***^ | -1.99^***^ | -2.12^***^ | -2.09^***^ |
| (Ref. Treatment) | (0.09) | (0.10) | (0.15) | (0.14) | (0.24) | (0.10) | (0.10) | (0.09) |
| Invasive (Ref. | 0.01 | -0.21^**^ | -0.22^***^ | -0.21^**^ | -0.22^**^ | -0.21^**^ | -0.21^**^ | -0.21^**^ |
| Noninvasive) | (0.09) | (0.07) | (0.07) | (0.07) | (0.07) | (0.07) | (0.07) | (0.07) |
| Morality first | 0.10 | 0.10 | 0.10 | 0.10 | 0.09 | 0.10 | 0.10 | 0.10 |
| (Ref. Second) | (0.07) | (0.10) | (0.07) | (0.07) | (0.07) | (0.07) | (0.07) | (0.07) |
| Stress | 0.12^*^ | 0.11^*^ | -0.04 | 0.11^*^ | 0.11^*^ | 0.11^*^ | 0.11^*^ | 0.11^*^ |
|  | (0.06) | (0.06) | (0.07) | (0.06) | (0.06) | (0.06) | (0.06) | (0.06) |
| Low cognitive | -0.07 | -0.07 | -0.07 | -0.21^**^ | -0.07 | -0.06 | -0.06 | -0.06 |
| functioning | (0.06) | (0.06) | (0.06) | (0.08) | (0.06) | (0.06) | (0.06) | (0.06) |
| Age | -0.00 | -0.00 | -0.00 | -0.00 | 0.00 | -0.00 | -0.00 | -0.00 |
|  | (0.00) | (0.00) | (0.00) | (0.00) | (0.00) | (0.00) | (0.00) | (0.00) |
| Religiosity | -0.02^*^ | -0.02^*^ | -0.03^*^ | -0.03^*^ | -0.02^*^ | -0.00 | -0.02^*^ | -0.02^*^ |
|  | (0.01) | (0.01) | (0.01) | (0.01) | (0.01) | (0.01) | (0.01) | (0.01) |
| Secondary education | 0.15^*^ | 0.14^*^ | 0.15^*^ | 0.14^*^ | 0.13 | 0.14^*^ | 0.17 | 0.15^*^ |
| (Ref. Lower) | (0.07) | (0.07) | (0.07) | (0.07) | (0.07) | (0.07) | (0.10) | (0.07) |
| Female | -0.03 | -0.03 | -0.04 | -0.05 | -0.03 | -0.03 | -0.03 | 0.04 |
| (Ref. Male) | (0.07) | (0.07) | (0.07) | (0.07) | (0.07) | (0.07) | (0.07) | (0.10) |
| Enhancement× | -0.44^**^ |  |  |  |  |  |  |  |
| Invasive | (0.13) |  |  |  |  |  |  |  |
| Enhancement× |  | -0.00 |  |  |  |  |  |  |
| Morality first |  | (0.13) |  |  |  |  |  |  |
| Enhancement× |  |  | 0.30^***^ |  |  |  |  |  |
| Stress |  |  | (0.09) |  |  |  |  |  |
| Enhancement× |  |  |  | 0.29^**^ |  |  |  |  |
| Low cog. funct. |  |  |  | (0.10) |  |  |  |  |
| Enhancement× |  |  |  |  | -0.01^**^ |  |  |  |
| Age |  |  |  |  | (0.00) |  |  |  |
| Enhancement× |  |  |  |  |  | -0.05^*^ |  |  |
| Religiosity |  |  |  |  |  | (0.02) |  |  |
| Enhancement× |  |  |  |  |  |  | -0.06 |  |
| Secondary education |  |  |  |  |  |  | (0.14) |  |
| Enhancement× |  |  |  |  |  |  |  | -0.15 |
| Female |  |  |  |  |  |  |  | (0.14) |
| Anonymity | -0.02 | -0.02 | -0.02 | -0.03 | -0.02 | -0.02 | -0.02 | -0.02 |
| perceptions | (0.05) | (0.05) | (0.05) | (0.05) | (0.05) | (0.05) | (0.05) | (0.05) |
| Constant | 3.25^***^ | 3.39^***^ | 3.59^***^ | 3.59^***^ | 3.04^***^ | 3.28^***^ | 3.38^***^ | 3.35^***^ |
|  | (0.30) | (0.30) | (0.30) | (0.30) | (0.31) | (0.30) | (0.30) | (0.30) |
| *F-test* | 99.83^***^ | 97.90^***^ | 99.90^***^ | 99.34^***^ | 99.80^***^ | 98.85^***^ | 97.93^***^ | 98.13^***^ |

*Notes:* *B*-coefficients (standard errors in brackets); ^*^*p*<0.05, ^**^*p*<0.01, ^***^*p*<0.001. ^a^*N* lower than in Table 2 due to the missing values in the anonymity variable.

## **Table S7:** Ordinary least regression of the use willingness regarding BSDs – testing for effects of purpose of use (enhancement vs. treatment) conditional on each study variable (*N_Experiment 1_*=1,083^a^) – controlled for anonymity perceptions.

|  | M_1_ | M_2_ | M_3_ | M_4_ | M_5_ | M_6_ | M_7_ | M_8_ |
| --- | --- | --- | --- | --- | --- | --- | --- | --- |
| Enhancement | -2.15^***^ | -2.05^***^ | -2.63^***^ | -2.58^***^ | -1.80^***^ | -2.19^***^ | -2.16^***^ | -2.26^***^ |
| (Ref. Treatment) | (0.09) | (0.09) | (0.14) | (0.13) | (0.22) | (0.09) | (0.09) | (0.08) |
| Invasive (Ref. | -0.21^*^ | -0.28^***^ | -0.29^***^ | -0.28^***^ | -0.28^***^ | -0.28^***^ | -0.28^***^ | -0.28^***^ |
| Noninvasive) | (0.09) | (0.06) | (0.06) | (0.06) | (0.06) | (0.06) | (0.06) | (0.06) |
| Morality first | 0.20^**^ | 0.37^***^ | 0.20^**^ | 0.20^**^ | 0.20^**^ | 0.20^**^ | 0.20^**^ | 0.20^**^ |
| (Ref. Second) | (0.06) | (0.09) | (0.06) | (0.06) | (0.06) | (0.06) | (0.06) | (0.06) |
| Stress | 0.08 | 0.08 | -0.05 | 0.08 | 0.08 | 0.08 | 0.08 | 0.08 |
|  | (0.05) | (0.05) | (0.07) | (0.05) | (0.05) | (0.05) | (0.05) | (0.05) |
| Low cognitive | -0.06 | -0.06 | -0.06 | -0.22^**^ | -0.06 | -0.06 | -0.06 | -0.06 |
| functioning | (0.06) | (0.06) | (0.06) | (0.07) | (0.06) | (0.06) | (0.06) | (0.06) |
| Age | -0.01^**^ | -0.01^**^ | -0.01^**^ | -0.01^**^ | -0.00 | -0.01^**^ | -0.01^**^ | -0.01^**^ |
|  | (0.00) | (0.00) | (0.00) | (0.00) | (0.00) | (0.00) | (0.00) | (0.00) |
| Religiosity | -0.02 | -0.01 | -0.02 | -0.02 | -0.02 | -0.01 | -0.02 | -0.02 |
|  | (0.01) | (0.01) | (0.01) | (0.01) | (0.01) | (0.01) | (0.01) | (0.01) |
| Secondary education | 0.05 | 0.05 | 0.05 | 0.04 | 0.04 | 0.05 | 0.11 | 0.05 |
| (Ref. Lower) | (0.07) | (0.06) | (0.06) | (0.06) | (0.06) | (0.07) | (0.09) | (0.07) |
| Female | -0.10 | -0.10 | -0.11 | -0.12 | -0.10 | -0.10 | -0.10 | -0.14 |
| (Ref. Male) | (0.07) | (0.07) | (0.07) | (0.07) | (0.07) | (0.07) | (0.07) | (0.09) |
| Enhancement× | -0.15 |  |  |  |  |  |  |  |
| Invasive | (0.13) |  |  |  |  |  |  |  |
| Enhancement× |  | -0.34^**^ |  |  |  |  |  |  |
| Morality first |  | (0.13) |  |  |  |  |  |  |
| Enhancement× |  |  | 0.28^**^ |  |  |  |  |  |
| Stress |  |  | (0.08) |  |  |  |  |  |
| Enhancement× |  |  |  | 0.30^**^ |  |  |  |  |
| Low cog. funct. |  |  |  | (0.10) |  |  |  |  |
| Enhancement× |  |  |  |  | -0.01^*^ |  |  |  |
| Age |  |  |  |  | (0.00) |  |  |  |
| Enhancement× |  |  |  |  |  | -0.01 |  |  |
| Religiosity |  |  |  |  |  | (0.02) |  |  |
| Enhancement× |  |  |  |  |  |  | -0.11 |  |
| Secondary education |  |  |  |  |  |  | (0.13) |  |
| Enhancement× |  |  |  |  |  |  |  | 0.07 |
| Female |  |  |  |  |  |  |  | (0.13) |
| Anonymity | -0.01 | -0.00 | -0.00 | -0.01 | -0.01 | -0.01 | -0.01 | -0.01 |
| perceptions | (0.05) | (0.05) | (0.05) | (0.05) | (0.05) | (0.05) | (0.05) | (0.05) |
| Constant | 3.05^***^ | 3.01^***^ | 3.28^***^ | 3.31^***^ | 2.90^***^ | 3.07^***^ | 3.08^***^ | 3.12^***^ |
|  | (0.28) | (0.28) | (0.28) | (0.28) | (0.29) | (0.28) | (0.28) | (0.28) |
| *F-test* | 119.53^***^ | 120.71^***^ | 121.44^***^ | 121.25^***^ | 120.05^***^ | 119.31^***^ | 119.41^***^ | 119.32^***^ |

*Notes:* *B*-coefficients (standard errors in brackets); ^*^*p*<0.05, ^**^*p*<0.01, ^***^*p*<0.001. ^a^*N* lower than in Table 2 due to the missing values in the anonymity variable.

## **Table S8:** Ordinary least regression of the moral acceptability regarding BCIs – testing for effects of purpose of use (enhancement vs. treatment) conditional on each study variable (*N_Experiment 2_*=1,080^a^) – controlled for anonymity perceptions.

|  | M_1_ | M_2_ | M_3_ | M_4_ | M_5_ | M_6_ | M_7_ | M_8_ |
| --- | --- | --- | --- | --- | --- | --- | --- | --- |
| Enhancement | -1.90^***^ | -2.03^***^ | -2.16^***^ | -2.05^***^ | -1.46^***^ | -1.76^***^ | -1.86^***^ | -1.78^***^ |
| (Ref. Treatment) | (0.10) | (0.10) | (0.16) | (0.15) | (0.27) | (0.11) | (0.10) | (0.10) |
| Invasive (Ref. | -0.34^***^ | -0.36^***^ | -0.36^***^ | -0.36^***^ | -0.36^***^ | -0.36^***^ | -0.36^***^ | -0.37^***^ |
| Noninvasive) | (0.10) | (0.07) | (0.07) | (0.07) | (0.07) | (0.07) | (0.07) | (0.07) |
| Morality first | 0.18^*^ | 0.07 | 0.18^*^ | 0.18^*^ | 0.18^*^ | 0.18^*^ | 0.18^*^ | 0.18^*^ |
| (Ref. Second) | (0.07) | (0.10) | (0.07) | (0.07) | (0.07) | (0.07) | (0.07) | (0.07) |
| Stress | -0.03 | -0.03 | -0.11 | -0.03 | -0.03 | -0.03 | -0.03 | -0.03 |
|  | (0.06) | (0.06) | (0.07) | (0.06) | (0.06) | (0.06) | (0.06) | (0.06) |
| Low cognitive | 0.07 | 0.07 | 0.07 | 0.01 | 0.07 | 0.07 | 0.07 | 0.07 |
| functioning | (0.06) | (0.06) | (0.06) | (0.08) | (0.06) | (0.06) | (0.06) | (0.06) |
| Age | -0.01^***^ | -0.01^***^ | -0.01^***^ | -0.01^***^ | -0.01 | -0.01^***^ | -0.01^***^ | -0.01^***^ |
|  | (0.00) | (0.00) | (0.00) | (0.00) | (0.00) | (0.00) | (0.00) | (0.00) |
| Religiosity | -0.02^*^ | -0.02^*^ | -0.02^*^ | -0.02^*^ | -0.02^*^ | -0.00 | -0.02^*^ | -0.02^*^ |
|  | (0.01) | (0.01) | (0.01) | (0.01) | (0.01) | (0.02) | (0.01) | (0.01) |
| Secondary education | 0.11 | 0.11 | 0.11 | 0.11 | 0.12 | 0.12 | 0.17 | 0.11 |
| (Ref. Lower) | (0.07) | (0.07) | (0.07) | (0.07) | (0.07) | (0.07) | (0.10) | (0.07) |
| Female | -0.17^*^ | -0.17^*^ | -0.17^*^ | -0.17^*^ | -0.17^*^ | -0.17^*^ | -0.17^*^ | -0.02 |
| (Ref. Male) | (0.07) | (0.07) | (0.07) | (0.07) | (0.07) | (0.07) | (0.07) | (0.10) |
| Enhancement× | -0.04 |  |  |  |  |  |  |  |
| Invasive | (0.14) |  |  |  |  |  |  |  |
| Enhancement× |  | 0.22 |  |  |  |  |  |  |
| Morality first |  | (0.14) |  |  |  |  |  |  |
| Enhancement× |  |  | 0.16 |  |  |  |  |  |
| Stress |  |  | (0.09) |  |  |  |  |  |
| Enhancement× |  |  |  | 0.11 |  |  |  |  |
| Low cog. funct. |  |  |  | (0.11) |  |  |  |  |
| Enhancement× |  |  |  |  | -0.01 |  |  |  |
| Age |  |  |  |  | (0.00) |  |  |  |
| Enhancement× |  |  |  |  |  | -0.05^*^ |  |  |
| Religiosity |  |  |  |  |  | (0.02) |  |  |
| Enhancement× |  |  |  |  |  |  | -0.11 |  |
| Secondary education |  |  |  |  |  |  | (0.14) |  |
| Enhancement× |  |  |  |  |  |  |  | -0.31^*^ |
| Female |  |  |  |  |  |  |  | (0.14) |
| Anonymity | 0.02 | 0.02 | 0.02 | 0.02 | 0.02 | 0.02 | 0.02 | 0.02 |
| perceptions | (0.05) | (0.05) | (0.05) | (0.05) | (0.05) | (0.05) | (0.05) | (0.05) |
| Constant | 3.76^***^ | 3.83^***^ | 3.90^***^ | 3.84^***^ | 3.55^***^ | 3.67^***^ | 3.73^***^ | 3.68^***^ |
|  | (0.30) | (0.30) | (0.31) | (0.31) | (0.33) | (0.30) | (0.30) | (0.30) |
| *F-test* | 73.56^***^ | 73.92^***^ | 74.02^***^ | 73.72^***^ | 74.03^***^ | 74.22^***^ | 73.64^***^ | 74.30^***^ |

*Notes:* *B*-coefficients (standard errors in brackets); ^*^*p*<0.05, ^**^*p*<0.01, ^***^*p*<0.001. ^a^*N* lower than in Table 2 due to the missing values in the anonymity variable.

## **Table S9:** Ordinary least regression of the use willingness regarding BCIs – testing for effects of purpose of use (enhancement vs. treatment) conditional on each study variable (*N_Experiment 2_*=1,080^a^) – controlled for anonymity perceptions.

|  | M_1_ | M_2_ | M_3_ | M_4_ | M_5_ | M_6_ | M_7_ | M_8_ |
| --- | --- | --- | --- | --- | --- | --- | --- | --- |
| Enhancement | -2.11^***^ | -1.96^***^ | -2.35^***^ | -2.25^***^ | -1.67^***^ | -1.97^***^ | -2.05^***^ | -1.97^***^ |
| (Ref. Treatment) | (0.10) | (0.10) | (0.15) | (0.14) | (0.26) | (0.10) | (0.10) | (0.09) |
| Invasive (Ref. | -0.39^***^ | -0.36^***^ | -0.36^***^ | -0.35^***^ | -0.35^***^ | -0.36^***^ | -0.36^***^ | -0.36^***^ |
| Noninvasive) | (0.10) | (0.07) | (0.07) | (0.07) | (0.07) | (0.07) | (0.07) | (0.07) |
| Morality first | 0.21^**^ | 0.33^***^ | 0.20^**^ | 0.21^**^ | 0.21^**^ | 0.21^**^ | 0.21^**^ | 0.21^**^ |
| (Ref. Second) | (0.07) | (0.10) | (0.07) | (0.07) | (0.07) | (0.07) | (0.07) | (0.07) |
| Stress | 0.08 | 0.09 | -0.01 | 0.08 | 0.08 | 0.08 | 0.08 | 0.08 |
|  | (0.05) | (0.05) | (0.07) | (0.05) | (0.05) | (0.05) | (0.05) | (0.05) |
| Low cognitive | -0.02 | -0.03 | -0.03 | -0.10 | -0.02 | -0.03 | -0.03 | -0.02 |
| functioning | (0.06) | (0.06) | (0.06) | (0.08) | (0.06) | (0.06) | (0.06) | (0.06) |
| Age | -0.01^***^ | -0.01^***^ | -0.01^***^ | -0.01^***^ | -0.01^**^ | -0.01^***^ | -0.01^***^ | -0.01^***^ |
|  | (0.00) | (0.00) | (0.00) | (0.00) | (0.00) | (0.00) | (0.00) | (0.00) |
| Religiosity | -0.01 | -0.01 | -0.01 | -0.01 | -0.01 | 0.00 | -0.01 | -0.01 |
|  | (0.01) | (0.01) | (0.01) | (0.01) | (0.01) | (0.01) | (0.01) | (0.01) |
| Secondary education | 0.09 | 0.09 | 0.09 | 0.09 | 0.09 | 0.09 | 0.12 | 0.09 |
| (Ref. Lower) | (0.07) | (0.07) | (0.07) | (0.07) | (0.07) | (0.07) | (0.10) | (0.07) |
| Female | -0.21^**^ | -0.21^**^ | -0.21^**^ | -0.22^**^ | -0.21^**^ | -0.21^**^ | -0.21^**^ | -0.10 |
| (Ref. Male) | (0.07) | (0.07) | (0.07) | (0.07) | (0.07) | (0.07) | (0.07) | (0.10) |
| Enhancement× | 0.07 |  |  |  |  |  |  |  |
| Invasive | (0.13) |  |  |  |  |  |  |  |
| Enhancement× |  | -0.23 |  |  |  |  |  |  |
| Morality first |  | (0.13) |  |  |  |  |  |  |
| Enhancement× |  |  | 0.18^*^ |  |  |  |  |  |
| Stress |  |  | (0.09) |  |  |  |  |  |
| Enhancement× |  |  |  | 0.15 |  |  |  |  |
| Low cog. funct. |  |  |  | (0.10) |  |  |  |  |
| Enhancement× |  |  |  |  | -0.01 |  |  |  |
| Age |  |  |  |  | (0.00) |  |  |  |
| Enhancement× |  |  |  |  |  | -0.03 |  |  |
| Religiosity |  |  |  |  |  | (0.02) |  |  |
| Enhancement× |  |  |  |  |  |  | -0.06 |  |
| Secondary education |  |  |  |  |  |  | (0.14) |  |
| Enhancement× |  |  |  |  |  |  |  | -0.23 |
| Female |  |  |  |  |  |  |  | (0.13) |
| Anonymity | 0.01 | 0.02 | 0.01 | 0.01 | 0.01 | 0.02 | 0.01 | 0.02 |
| perceptions | (0.05) | (0.05) | (0.05) | (0.05) | (0.05) | (0.05) | (0.05) | (0.05) |
| Constant | 3.59^***^ | 3.50^***^ | 3.72^***^ | 3.66^***^ | 3.38^***^ | 3.51^***^ | 3.55^***^ | 3.50^***^ |
|  | (0.29) | (0.29) | (0.29) | (0.29) | (0.31) | (0.29) | (0.29) | (0.29) |
| *F-test* | 96.27^***^ | 96.76^***^ | 96.98^***^ | 96.61^***^ | 96.70^***^ | 96.58^***^ | 96.25^***^ | 96.74^***^ |

*Notes:* *B*-coefficients (standard errors in brackets); ^*^*p*<0.05, ^**^*p*<0.01, ^***^*p*<0.001. ^a^*N* lower than in Table 2 due to the missing values in the anonymity variable.
